# Demonstration of strain-specific CD8 T cell responses to *Theileria annulata*

**DOI:** 10.1111/j.1365-3024.2008.01038.x

**Published:** 2008-08

**Authors:** N D Machugh, A C Burrells, W I Morrison

**Affiliations:** Division of Veterinary Clinical Sciences, Royal (Dick) School of Veterinary Studies, University of EdinburghEaster Bush Campus, Roslin, Midlothian, UK

**Keywords:** bovine CD8, cytotoxic T cell, strain specificity, *Theileria*

## Abstract

The present study set out to examine the nature and specificity of the bovine CD8 T cell response at the clonal level in a group of eight animals immunized with a cloned population of *Theileria annulata*. The results demonstrated that immunized animals generated parasite-specific CD8 T cells that produced IFNγ in response to parasite stimulation but had highly variable levels of cytotoxicity for parasitized cells. The study also demonstrated that these parasite-specific CD8 T cells could be propagated and cloned *in vitro* from the memory T cell pool of cattle immunized with live *T. annulata* parasites. Within the small group of animals studied, there was evidence that responses were preferentially directed to antigens presented by an A10^+^ class I major histocompatibility complex (MHC) haplotype, suggesting that responses restricted by products of this haplotype may be dominant. The A10-restricted responses showed differential recognition of different parasite isolates and clones. By using a cloned population of parasites both for immunization of the animals and for *in vitro* analyses of the responses, we obtained unambiguous evidence that at least a proportion of CD8 T cells restricted by one MHC haplotype were parasite strain restricted.

## Introduction

The apicomplexan protozoan parasite *Theileria annulata* causes an acute, sometimes fatal lymphoproliferative disease in cattle known as tropical theileriosis, which results in serious economic losses over a large area extending from the Mediterranean basin to India and parts of Asia. Similar to Plasmodium species, *T. annulata* undergoes successive development in nucleated cells and erythrocytes, but, unlike malaria, disease results from multiplication within nucleated cells, which can occur in macrophages, dendritic cells and B lymphocytes (1,2). In common with other apicomplexa, including malaria parasites, immunity to *T. annulata* is believed to be mediated by T cell responses directed against parasitized cells. Cattle can be immunized by administration of parasitized cells that have been attenuated by prolonged *in vitro* passage (3) or by infection with sporozoites and concomitant treatment with long acting tetracycline (4). Cattle immunized by these methods have been shown to generate both CD4 and CD8 T cell responses specific for parasitized leucocytes (5–8).

Attenuated parasitized cell lines have been used successfully to vaccinate cattle in the field (9). However, because of a reluctance to introduce live parasites originating from other countries, vaccines used in each country have in most cases been generated independently from locally isolated parasite strains. Immunization with such vaccines appears to provide a high level of protection against field challenge, although there are reports of some immunized animals developing clinical disease following challenge (3). Moreover, experimental studies have shown that, while immunized animals are solidly immune to challenge with the (homologous) parasite isolate from which the vaccine was derived, the levels of protection against challenge with heterologous isolates are less consistent (9,10). The immunological basis of this incomplete cross-protection is not understood, although studies of a related parasite, *Theileria parva*, have provided evidence that failure of strains to cross-protect is associated with recognition of polymorphic parasite antigens by CD8 T cell responses (11,12).

Previous studies of CD8^+^ T cell responses to *T. annulata* have focused mainly on direct analyses of the response in animals undergoing immunization or challenge, when effector cells are present in sufficient numbers for detection by direct cytotoxicity assays. These studies have shown that the emergence of detectable major histocompatibility complex (MHC)-restricted T cell-mediated cytotoxicity coincides with clearance of the parasite, suggesting that this response is involved in control of the infection (13). One study involving a set of identical twin calves reported evidence that the cytotoxic response in the immunized twin was parasite strain specific (7). Further detailed analyses of the phenotype and specificity of the responding cells has been hampered by difficulties in establishing and maintaining parasite-specific T cell lines with cytotoxic activity. Genetic and antigenic heterogeneity within parasite isolates represents an additional potential complicating factor in the interpretation of data from studies of antigenic specificity (14,15). Analyses of isoenzymes and the use of genomic probes have provided evidence of heterogeneity within isolates of *T. annulata* (10) and this has been confirmed using a recently developed panel of DNA satellite markers (16). Parasitized cell lines derived from such isolates are likely to contain varying proportions of the genetically different components of the isolate. Moreover, typing of cell lines at different levels of *in vitro* passage has shown that some of the components of an isolate can be lost with repeated passage (10). These observations highlight the need to use defined populations of parasites for any studies of parasite strain specificity of T cell responses. The recent production of sporozoites from a cloned *T. annulata* parasite (17), coupled with the availability of molecular genotyping techniques, now provide the necessary tools to help dissect the antigenic specificity of T cell responses to *T. annulata*.

The present study set out to examine the nature and specificity of the CD8 T cell response at the clonal level in animals immunized with a cloned population of *T. annulata.* The results demonstrate that immunized animals generate parasite-specific CD8 T cells that produce IFNγ in response to parasite stimulation but have highly variable levels of cytotoxicity for parasitized cells. Within the group of animals studied, responses restricted by an A10^+^ class I MHC haplotype were found to be highly dominant and showed differential recognition of different parasite clones.

## Materials and methods

### *Theileria annulata*-immune animals

Clinically normal castrated male Friesian/Holstein (*Bos taurus*) cattle were used throughout these studies. Animals carrying defined MHC haplotypes, from which the expressed class I genes have been identified and cloned, were selected for the study. Animals were typed for class I antigens using a combination of monoclonal antibodies and PCR/sequencing methods (18,19).

Eight animals were immunized with a cloned population of *T. annulata* (C9) derived from the Ankara isolate (17,20). Four of the animals were immunized by inoculation with sporozoites from a cryopreserved stabilate and simultaneous treatment with long-acting oxytetracycline; the remaining four animals were immunized by subcutaneous administration of 5 × 10^6^ allogeneic *T. annulata*–infected cells from a cell line infected with C9 (4). Animals were challenged 1–3 months later with a lethal dose of sporozoite stabilate, five animals with C9, 1 with the Gharb isolate, and the remaining two with the Hissar stock (Table 1).

**Table 1:** Cytotoxic activity of CD8^+^ T cell clones derived from eight *T. annulata* immune animals on autologous *T. annulata*– infected targets

				Clones showing specific cytotoxicity (≥ 5%)		
				
Animal	MHC type^a^	Immunization/challenge^b^	T cell clones	Number (%)	Range of killing (%)	Mean (%)
216	(A15/nk)	C9 cell line/Hissar	82	42 (51)	5–32	20
219	(A10/A19)	C9 cell line/Hissar	65	43 (66)	5–60	25
633	(A18/A31)	C9 sporoz./C9	42	14 (33)	6–36	14
663	(A14/A18)	C9 sporoz./C9	42	3 (7)	8–51	23
744	(A10/A20)	C9 sporoz./C9	36	27 (75)	15–95	67
987	(A14/A19)	C9 sporoz./C9	42	3 (7)	5–5·2	5
1147	(A10/A15)	C9 cell line/C9	90	65 (72)	5–73	39
1157	(A14/A19)	C9 cell line/Gharb	96	15 (16)	5–65	17

a nk = not known.

b All animals were immunized with a cloned population of *T. annulata* (C9), either by infection with sporozoites (sporoz.) and simultaneous treatment with long-acting tetracycline or by administration of allogeneic parasitized cells. The animals were subsequently challenged with sporozoites, five with C9, two with the Hissar stock and one with the Gharb stock.

### *Theileria annulata*-infected cell lines and clones

Peripheral blood mononuclear cells (PBM) prepared by flotation on Ficoll-Paque (21) were infected *in vitro* with sporozoites from cryopreserved stocks of *T. annulata* as previously described (22). Cell lines were established with the Ankara, Gharb and Hissar uncloned isolates and the cloned C9 derivative of Ankara. These isolates are from geographically distinct locations: *T. annulata* Ankara was isolated in Turkey, *T. annulata* Gharb in Morocco and *T. annulata* Hissar in India. They also have been shown to differ genotypically based on analyses with satellite DNA markers (16). Cloned infected cell lines were derived from the Gharb and Hissar isolates by limiting dilution cloning of bulk cell lines infected with the different isolates. All lines were maintained in RPMI 1640 medium containing 10 mm HEPES buffer, supplemented with 10% heat-inactivated foetal bovine serum, 5 × 10^−5^ m2-mercaptoethanol, 2 mm l-glutamine and 50 mg/mL Pen/Strep (RPMI culture medium). Cell lines infected with the Muguga stock of *T. parva* were used in some experiments; these were prepared as described for *T. annulata*.

### Flow cytometry

Suspensions of viable cells were stained by indirect immunofluorescence with monoclonal antibodies specific for bovine leucocyte surface markers and analysed on a FACScan II analyser (Becton Dickinson, UK). Antibodies specific for the following surface antigens were used in this study: CD3 – MM1A (IgG1 23); CD8 – IL-A51 (IgG1 22); CD4 – IL-A12 (IgG2a) (24); WC1, a 215 kDa antigen expressed on the surface of CD4^−^CD8^−^ bovine γδ T cells – IL-A29 (IgG1) and CC15 (IgG2a) (25); bovine γδ T cell receptor – GB21A (IgG2b) (25).

### Generation of CD8^+^ T cell clones

Parasite-specific CD8 T cell clones were generated essentially as previously described for *T. parva* (21). Briefly, PBM cells from *T. annulata*-immune animals were plated out in 24 well tissue culture plates at 4 × 10^6^ cells per well with 2 × 10^5^ γ-irradiated *T. annulata*-infected stimulator cells in a total volume of 2 mL of RPMI culture medium per well. In seven of the eight animals studied an autologous parasitized cell line was used as a stimulator. Stimulator cells infected with the C9 clone of *T. annulata* were used for all animals. An autologous line was not available for one animal (744) and therefore an MHC-homozygous line carrying one of the haplotypes (A10) expressed by this animal was employed for stimulation. After 7 days, cells were harvested and re-stimulated with irradiated autologous stimulators as in the primary culture. After a further 7 days, the cultures were enriched for CD8 T cells by removal of CD4 and γδ T cells by complement-mediated killing with mAb IL-A12 (CD4) and CC15 (γδ T cells). Normal rabbit serum at a dilution of 1 : 3 was used as a source of complement.

The remaining T cells were incubated overnight in RPMI culture medium containing 100 units per ml of recombinant human IL-2 (Chiron Ltd, UK). The following day the cells were cloned by limiting dilution in 96-well round bottom tissue culture plates using 1 × 10^3^ γ-irradiated autologous *T. annulata*-infected cells as stimulators and 2 × 10^4^ autologous irradiated PBM as fillers per well, in a final volume of 200 µL of RPMI culture medium containing 100 units/mL of recombinant human IL-2.

Two weeks later, cells from wells showing good growth were transferred to 48-well plates and expanded by re-stimulating with 2·5 × 10^5^ irradiated autologous *T. annulata*-infected cells and 100 units of recombinant human IL-2 per well in a final volume of 1 mL. In order to improve the likelihood that the cell populations were true clones, cells were lifted from plates with wells showing growth at clonal or near-clonal frequency, that is, plates with growth in less than 30% of wells (21).

The phenotype of the clones was determined by mAb staining and flow cytometry.

### Cytotoxicity assay

A 4-h Indium oxine [^111^In] (Amersham Medical, UK) release assay was used to assess the cytotoxicity of expanded CD8 T cell clones as previously described (21). Briefly, for screening of clones, 100 µL of cells were removed from each well of the 48-well plates and incubated with 5 × 10^3 111^In-labelled *T. annulata*-infected target cells in V-bottom 96-well plates. In subsequent assays, defined numbers of effector cells were used at a range of effector to target ratios. Plates were incubated at 37°C for 4 h in a humidified atmosphere of 5% CO_2_ in air. Maximum ^111^In release was measured by incubating target cells in 0·1% Tween in H_2_O for 4 h and spontaneous (background) release was measured by incubating targets in RPMI growth medium without effectors. Radioactivity in supernatants was measured using a γ counter and percent cytotoxicity was calculated as 100× (test release-spontaneous release)/maximum release-spontaneous release).

### Interferon γ (IFNγ) assay

Antigen recognition by CD8 T cell clones was also assayed by detection of IFNγ release following antigenic stimulation, using a biological assay as described (26). Briefly, 5–10 × 10^3^ bovine CD8 T cells were incubated with 5 × 10^4^ *Theileria*-infected target cells in 200 µL of RPMI growth medium containing 100 U rHumIL-2, in 96-well round bottom plates at 37°C for 24–48 h. Supernatants were collected and bovine IFNγ activity determined by measuring up-regulation of MHC class II on bovine endothelial cells. 100 µL of each supernatant were added to bovine endothelial cells plated out in 96-well flat-bottom plates at 2 × 10^4^ cells per well. 24–48 h later the endothelial cells were lifted with 0·25% trypsin/EDTA (Invitrogen, UK) and the MHC class II levels were assessed by staining with an antibovine class II DR mAb IL-A21 (27) followed by FITC-labelled antimouse Ig (Sigma). Results were expressed as percentage MHC class II positive bovine endothelial cells.

## Results

### Parasite-specific CD8 T cell lines show variable levels of cytotoxicity

Eight animals were immunized with *T. annulata* C9 and were shown to be solidly immune when challenged with the same parasite one month later (data not shown). T cell lines were generated using PBM collected following challenge. Phenotypic analyses of the cultures after two rounds of *in-vitro* stimulation with irradiated parasitized cells showed that > 90% of the cells were T cells (CD3^+^) and that a large majority of them had a CD4 phenotype (40–80%). The cultures contained smaller numbers of both CD8^+^ cells (16–27%) and γδ T cells (5–23%).

**Figure 1:**
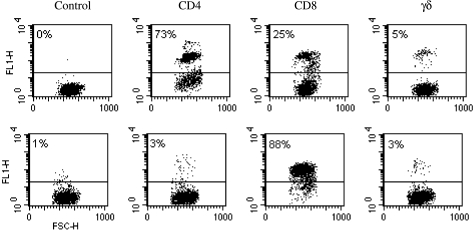
(Top) FACS analysis of PBM from animal 744 after 2 rounds of re-stimulation with a *T. annulata* Ankara (C9) infected A10 homozygous cell line prior to enrichment of the CD8 component by mAb and complement mediated lysis. (Lower) FACS analysis of PBM after lysis of the CD4 and γδ populations showing enrichment of the CD8 population from 25 to 88%. The Y axis shows log of fluorescence height and the X-axis shows forward scatter. Percentage of positive cells for each antibody is shown in the upper left hand corner of each graph.

Complement mediated killing of CD4^+^ and γδ T cells resulted in enrichment of the CD8^+^ population to > 80% (Figure 1). Cytotoxicity assays carried out with these bulk CD8 T cell-enriched cultures showed marked variation in the cytotoxic activity of the cultures, with levels ranging from 24% to 72% at effector target ratios of 80 : 1.

### Generation of CD8 T cell clones

Because of the possibility that clones within the CD8 T cell cultures were heterogeneous with respect to cytotoxicity and/or antigen specificity, the cultures were cloned by limiting dilution to allow further analyses. CD8^+^ T cell clones were successfully generated from all eight animals; between 36 and 96 clones were available for screening from each animal, depending on the success of the cloning.

### CD8 T cell clones show variable cytotoxic activity

**Figure 2:**
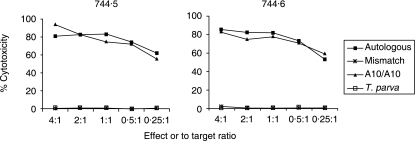
MHC restriction of CD8^+^ CTL clones 744·5 and 744·6. The cytotoxicity of two clones from animal 744 was analysed on autologous *T. annulata* and *T. parva* infected cell line targets, MHC – mismatched, and homozygous A10 *T. annulata* infected target cell lines.

The percentage of clones from each animal that had significant levels of cytotoxicity against the autologous parasitized target cell varied markedly, ranging from 7% to 75%. The levels of cytotoxicity of the positive clones from different animals also showed wide variation, the mean cytotoxicity for each animal ranging from 5% to 67% and levels of cytotoxicity of up to 95% were observed for individual clones (Table 1). In general, clones with the highest levels of cytotoxicity were found in those animals with the largest proportion of clones showing cytotoxic activity. A notable feature of these results was that the three cultures displaying the highest levels of cytotoxicity were all derived from animals bearing the A10 class I MHC specificity. The vast majority of these clones did not give significant levels of cytotoxicity when tested on an MHC-mismatched parasitized cell line and were therefore considered to be MHC-restricted (Figure 2). However, in six of the eight animals studied a variable number of clones (1–33%) were found to kill in a non MHC-restricted manner. These clones were not analysed further.

### CD8 T cell clones consistently produce IFNγ

**Figure 3:**
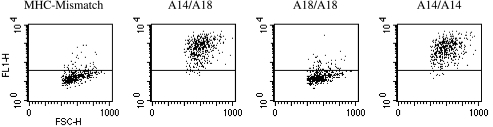
IFNγ release assay of MHC restriction of CD8^+^ clone 663·14: Dot plot FACS profiles of bovine endothelial cells stained with class II MHC-specific mAb IL-A21 showing a representative analysis of IFNγ release by a CD8^+^ clone. From left to right, cells cultured with supernatant from clone 663·14 incubated with an MHC-mismatch target, the autologous target, an A18 homozygous target, and an A14 homozygous target. All targets were infected with the *T. annulata* Ankara C9 stock.

A small subset of the clones exhibiting different levels of MHC-restricted cytotoxicity was also tested for their ability to produce IFNγ in response to stimulation with autologous or MHC-mismatched parasitized cells. All clones were found to produce IFNγ in an MHC-restricted manner regardless of their cytotoxic activity. Representative results are shown in Figure 3.

### CD8 T cell responses show a hierarchy in dominance of MHC restriction

To determine whether there was any bias in the MHC haplotype restriction of the CD8 T cell response, the specificity of a set of clones from six animals was tested using MHC-homozygous *T. annulata-*infected cell lines expressing each of the parental haplotypes. Clones were tested using either a cytotoxicity assay or, where the clones had low levels of cytotoxicity, an IFNγ assay. The results obtained from these six animals are summarized in Table 2. In all cases, there was a numerical bias in the response to one of the haplotypes. Although the study included only a small number of animals of each haplotype, there was an indication that the responses were directed towards certain haplotypes in preference to others. Thus in two animals carrying the A10 haplotype in combination with two different haplotypes, 39 of 40 clones and 14 of 15 clones were found to be A10-restricted; similarly 8 of 10 clones derived from each of two animals carrying the A14 haplotype were A14-restricted.

**Table 2:** MHC haplotype restriction of the CD8^+^ T cell response in cattle immunized with *Theileria annulata* Ankara (C9) isolate. The table shows the number of CD8^+^ clones restricted by each parental haplotype. (nk = unknown)

		Haplotype restriction						
		
Donor	MHC haplotype	A10	A14	A15	A18	A19	A31	other
216	A15/nk			5				22
219	A10/A19	39				1		
633	A18/A31				1		8	
663	A14/A18		8		2			
987	A14/A19		8			2		
1147	A10/A15	14		1				

### CD8 T cell clones display parasite strain specificity

**Figure 4:**
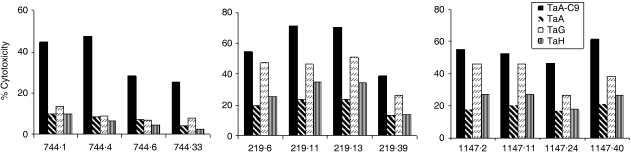
Cytotoxicity of a panel of 4 clones from animals 219, 744, and 1147 assayed on MHC –matched target cell lines infected with the cloned *T. annulata* stock C9 (TaA-C9) 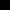, Uncloned Ankara isolate (TaA) 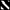 Gharb isolate (TaG) 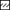, or Hissar isolate (TaH) 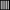.

Four CD8^+^ clones from each of the 3 A10^+^ animals (744, 219 and 1147) were analysed for parasite strain specificity using the Indium release cytotoxicity assay. The clones were first tested using target cell lines infected with the uncloned *T. annulata* isolates Ankara, Gharb or Hissar, as well as the cloned Ankara C9 stock. All 12 clones consistently showed higher levels of cytotoxicity on C9-infected targets than on targets infected with the uncloned *T. annulata* stocks, including the Ankara stock from which C9 was derived (Figure 4). Clones from one animal (744) exhibited a high level of specificity for the autologous C9-infected cell line, giving levels of cytotoxicity ranging from 25% to 47% against C9 compared to < 13% against the uncloned Ankara, Gharb and Hissar parasitized lines (Figure 4). Clones derived from the other two animals (219 and 1147) gave intermediate but variable levels of killing against targets infected with these uncloned *T. annulata* isolates (Figure 4).

**Figure 5:**
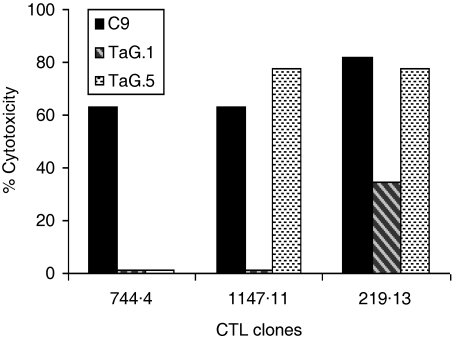
Cytotoxic activity of 3 CD8^+^ CTL clones tested on MHC-matched targets infected with the cloned *T. annulata* Ankara C9 parasite (C9) and two clones derived from a cell line infected with the Gharb isolate TaG.1 and TagG.5.

Because of the potentially heterogeneous make-up of the parasite population in the different isolates, the same panel of cloned CTL were tested on 4 cloned infected cell lines derived from the Gharb isolate. Representative results are shown in Figure 5. Three distinct patterns of reactivity were detected (1): CTL clones that recognized only targets infected with the cloned Ankara C9 parasite (2), clones that recognized one of the four cloned Gharb targets, and (3) CTL clones that recognized two of the cloned Gharb-infected targets (Figure 5). All four CTL clones from animal 744 displayed the first of these patterns, reflecting the high level of specificity of these clones for C9 when tested against the uncloned stocks. These results provide clear evidence of strain specificity of the response at the clonal level.

## Discussion

The present study has demonstrated that parasite-specific CD8 T cells can be propagated and cloned *in vitro* from the memory T cell pool of cattle immunized with live *T. annulata* parasites. Such clones displayed variable levels of cytolytic activity for parasitized cells but consistently produced IFNγ in response to antigenic stimulation. By using a cloned population of parasites both for immunization of the animals and for *in vitro* analyses of the responses, it was possible to obtain unambiguous evidence that at least a proportion of CD8 T cells restricted by one MHC haplotype were parasite strain restricted.

Previous studies of active T cell responses in animals undergoing immunization or challenge with *T. annulata* have demonstrated transient cytotoxic T cell responses in blood and lymph coinciding with clearance of the immunizing or challenge infection (7,13). Although the patterns of MHC restriction of these responses correlated with the class I phenotypes of the target cells tested, identification of the effectors as CD8 T cells was not formally demonstrated. Moreover, in contrast to experience with the related parasite *T. parva*, where the generation and long-term maintenance of cloned CD8^+^ T cells with cytolytic activity is well established (28,29), the successful generation and maintenance of CD8 T cell cloned lines from PBM of *T. annulata-*immune cattle has not been previously described. The results of the present study indicate that the use of cytotoxicity assays to detect a CD8 T cell response in such cultures is unreliable, since, although parasite-specific CD8 T cells were consistently generated in cultures derived from immune animals, their levels of cytolytic activity were sometimes very low. Studies of T cell responses to viruses in humans and mice have shown that the specific CD8 T cells are functionally heterogeneous at the clonal level, even within populations specific for the same epitope (30–32). Thus, variable proportions of clones produce IFNγ and are cytolytic and these proportions differ for responses to different pathogens. In the present study, all of the T cell clones tested produced detectable levels of IFNγ but the levels of cytotoxicity varied markedly between animals. An important issue is whether this variability reflects the true functional capacity of the memory cells or is a consequence of deficiencies in the culture systems used to maintain and assay the cells. The detection of cytotoxic T cell responses in immunized animals subjected to parasite challenge would indicate that CD8 T cells with cytolytic capacity have been retained in the memory pool following immunization. Hence the poor cytolytic activity in cultures from some immune cattle may be a consequence of either relative insensitivity of the *T. annulata*-infected target cells to lysis or an inability of the cells when used as stimulators to activate and maintain cytolytic function *in vitro*. The former possibility seems unlikely for the following reasons. First, in previous studies (5,7,13) a range of cell lines have been used successfully as target cells to detect the active cytotoxic T cell response *in vivo*. Second, the analyses of MHC restriction of our clones revealed similar levels of killing of the autologous and relevant MHC-matched targets. Third, we have found that *T. annulata-*infected cell lines, when used as antigen-presenting cells to present peptides to *T. parva-*specific cytotoxic CD8 T cells, are sensitive to lysis (N.D. MacHugh, T. Connelley and W.I. Morrison, unpublished data). These observations indicate that the variable cytotoxicity reflects the properties of the effector cells rather than the targets. A more plausible explanation is that the capacity of the parasitized cell lines to activate and maintain cytolytic function in the *in vitro* CD8 T cell cultures, when used as stimulator cells, may vary. Particularly intriguing was the finding that T cell clones derived from the three animals that generated a dominant A10-restricted CD8 T cell response showed the highest levels of killing. This may be due to the presence of a relative high density of the epitope(s) recognized by these T cells on the surface of the parasitized cells or the presence, within the T cell repertoires of these animals, of T cells with particularly high avidity receptors for these MHC-peptide combinations. Further studies will be required to address these issues.

Given the antigenic complexity of Theileria parasites and the fact that most bovine MHC haplotypes express at least two functional class I MHC genes (33), it might be expected that infection would induce strong CD8 T cell responses restricted by class I products of both MHC haplotypes. However, analyses of the MHC restriction of sets of T cell clones from each animal revealed a marked quantitative bias in restriction of the response to one haplotype. Moreover, although each haplotype was represented only in a few animals, the results indicated that responses restricted by certain haplotypes (notably those carrying A10 and A14) were dominant in comparison to others. The animals in this study had been subjected to immunization and challenge prior to analyses of responses and three of them had been challenged with a heterologous parasite stock. Hence, further studies will be required both to confirm that the haplotype dominance occurs following primary immunization and to determine whether the hierarchy of dominance is consistent in larger numbers of animals. Nevertheless, such preferential use of restriction elements is a commonly reported feature of CD8 T cell responses to viruses in humans and mice (34,35) and is also well documented for responses of cattle to *T. parva*, which are often focused entirely on antigens presented by one MHC haplotype (11,29). Furthermore, recent studies of responses of immune cattle to defined *T. parva* antigens have shown that a large component of the response is directed to one or two antigens (MacHugh, Connelley and Morrison, in preparation). The findings of the present study suggest that such focusing of the response on a limited number of dominant antigens may also be a feature of CD8 T cell responses to *T. annulata*, although confirmation of this must await identification of the target antigens.

As outlined earlier, the use of a cloned parasite (C9) in these studies ensured that there was no discrepancy between the parasite used to immunize the animals and that in the parasitized cell lines used for analyses of the immune responses. Although one of the 3 A10^+^ animals in which strain specificity of the response was investigated had also been subjected to challenge with a heterologous parasite stock (Hissar), all analyses of T cell responses utilized stimulator cells infected with C9. The strain specificity of a small subset of the CD8 T cell clones derived from the 3 A10^+^ animals was investigated, initially using uncloned parasites originally isolated from geographically distant sites, Morocco (Gharb), and India (Hissar), as well as the parent Turkisk isolate. The results of these experiments revealed consistently higher levels of killing of targets infected with C9 than those infected with the uncloned isolates including the Ankara isolate. This was particularly marked for clones from one of the animals (744) which showed a high degree of specificity for C9. These results suggested that the uncloned parasite isolates were antigenically heterogeneous with respect to the antigens recognized by these T cell clones. This was confirmed by analyses of four cloned derivatives of the Gharb isolate. The results demonstrated differential recognition of the Gharb parasite clones and revealed three distinct patterns of reactivity among the CD8 T cell clones tested, including clones that recognize only one or two of the four Gharb cloned parasites and others that recognized all four parasites. These findings provide clear evidence that at least a component of the A10-restricted CD8 T cell response in these animals is parasite strain-restricted, but that there is heterogeneity both of the strain specificity of CD8 T cells within the responding populations and of the target antigens within the uncloned parasite isolates. The results also imply that these T cells recognize several antigenic epitopes. Further studies are required using larger numbers of cloned parasites and CD8 T cell clones to obtain a more complete picture of the composition of the response in the individual animals.

While the precise role of CD8 T cell responses in immunity to *T. annulata* remains to be determined, the evidence that antigens recognized by parasite-specific CD8 T cells vary between parasite isolates and between cloned populations from the same isolate, provides a potential immunological basis for previously reported findings that cattle vaccinated with attenuated cell lines are not always protected against challenge with heterologous parasite isolates. Thus, as proposed by Dargouth *et al*. (10), early passage isolates of *T. annulata*, which are often genetically heterogeneous, are likely to contain sufficient antigenic diversity to induce T cell responses effective against most if not all parasite strains, whereas cell line vaccines that have undergone prolonged passage may be mono- or oligoclonal and therefore unable to induce a T cell response against a sufficiently diverse range of antigens to provide complete protection against all parasite strains. If indeed CD8 T cell responses play a critical role in immunity to *T. annulata*, the existence of variability in the target antigens also represents a potential obstacle to development of a subunit vaccine. Further studies are required, first to determine whether the strain-restricted nature of CD8 T cell responses described here for A10^+^ animals is also a feature of responses induced in animals of other MHC genotypes and second to attempt to identify and characterize the target antigens. The methodologies and cellular reagents developed in the course of the present study provide some of the necessary tools to address these objectives.

## References

[1] Howard CJ, Sopp P, Preston PM, Jackson LA, Brown CG (1993). Phenotypic analysis of bovine leukocyte cell lines infected with *Theileria annulata*. Vet Immunol Immunopathol.

[2] Spooner RL, Innes EA, Glass EJ, Brown CG (1989). *Theileria annulata* and *T. parva* infect and transform different bovine mononuclear cells. Immunology.

[3] Pipano E, Tsur I (1966). Experimental immunization against *Theileria annulata* with a tissue culture vaccine. Laboratory trials. Refuah Veterinarith.

[4] Pipano E, Samish M, Kriegel Y, Yeruham I (1981). Immunization of Friesian cattle against *Theileria annulata* by the infection-treatment method. Br Vet J.

[5] Innes EA, Millar P, Brown CG, Spooner RL (1989). The development and specificity of cytotoxic cells in cattle immunized with autologous or allogeneic *Theileria annulata*-infected lymphoblastoid cell lines. Parasite Immunol.

[6] Preston PM, Brown CG, Spooner RL (1983). Cell-mediated cytotoxicity in *Theileria annulata* infection of cattle with evidence for BoLA restriction. Clin Exp Immunol.

[7] Conze G, Campbell JDM, Nichani AK (1998). Evidence for strain specificity in cytotoxic T-lymphocyte-mediated, major histocompatibility complex class I-dependent killing of *Theileria annulata*-infected cells. Parasitol Res.

[8] Ahmed JS, Mehlhorn H (1999). Review: the cellular basis of the immunity to and immunopathogenesis of tropical theileriosis. Parasitol Res.

[9] Pipano E, Shkap V (2000). Vaccination against Tropical Theileriosis. Ann NY Acad Sci.

[10] Darghouth MA, Ben ML, Bouattour A (1996). A preliminary study on the attenuation of Tunisian schizont-infected cell lines of *Theileria annulata*. Parasitol Res.

[11] Taracha EL, Goddeeris BM, Teale AJ, Kemp SJ, Morrison WI (1995). Parasite strain specificity of bovine cytotoxic T cell responses to *Theileria parva* is determined primarily by immunodominance. J Immunol.

[12] Taracha EL, Goddeeris BM, Morzaria SP, Morrison WI (1995). Parasite strain specificity of precursor cytotoxic T cells in individual animals correlates with cross-protection in cattle challenged with *Theileria parva*. Infect Immun.

[13] Ahmed JS, Hartwig H, Schein E (1999). Generation of *Theileria annulata*-specific cytotoxic T lymphocytes coincides with the control of tropical theileriosis. Parasitol Res.

[14] Schnittger L, Katzer F, Biermann R (2002). Characterization of a polymorphic *Theileria annulata* surface protein (TaSP) closely related to PIM of *Theileria parva*: implications for use in diagnostic tests and subunit vaccines. Mol Biochem Parasitol.

[15] Gubbels MJ, Katzer F, Hide G, Jongejan F, Shiels BR (2000). Generation of a mosaic pattern of diversity in the major merozoite-piroplasm surface antigen of *Theileria annulata*. Mol Biochem Parasitol.

[16] Weir W, Ben-Miled L, Karagenc T (2007). Genetic exchange and sub-structuring in *Theileria annulata* populations. Mol Biochem Parasitol.

[17] Pain A, Renauld H, Berriman M (2005). Genome of the Host-Cell Transforming Parasite *Theileria annulata* Compared with *T. parva*. Science.

[18] Ellis SA, Staines KA, Stear MJ, Hensen EJ, Morrison WI (1998). DNA typing for BoLA class I using sequence-specific primers (PCR-SSP). Eur J Immunogenet.

[19] Ellis SA, Morrison WI, MacHugh ND (2005). Serological and molecular diversity in the cattle MHC class I region 2. Immunogenetics.

[20] Schein E (1975). On the life cycle of *Theileria annulata* (Dschunkowsky and Luhs, 1904) in the midgut and hemolymph of Hyalomma anatolicum excavatum (Koch, 1844). Z Parasitenkd.

[21] Goddeeris BM, Morrison WI (1988). Techniques for the generation, cloning, and characteization of bovine cytotoxic T cells specific for the protozoan *Theileria parva*. J Tissue Culture Meth.

[22] Brown CGD, Stagg RE, Punell GK (1973). Anhani & Kayane RC. Infection and transformation of bovine lymphoid cells *in vitro* by infective particles of *Theileri parva*. Nature.

[23] MacHugh ND, Mburu JK, Hamilton MJ, Davis WC (1998). Characterisation of a monoclonal antibody recognising the CD3epsilon chain of the bovine T cell receptor complex. Vet Immunol Immunopathol.

[24] Baldwin CL, Teale AJ, Naessens JG (1986). Characterization of a subset of bovine T lymphocytes that express BoT4 by monoclonal antibodies and function: similarity to lymphocytes defined by human T4 and murine L3T4 49. J Immunol.

[25] Clevers H, MacHugh ND, Bensaid A (1990). Identification of a bovine surface antigen uniquely expressed on CD4^−^CD8^−^ T cell receptor gamma/delta+ T lymphocytes 35. Eur J Immunol.

[26] Ballingall KT, Mwangi DM, MacHugh ND (2000). A highly sensitive, non-radioactive assay for T cell activation in cattle: applications in screening for antigens recognised by CD4 (+) and CD8 (+) T cells 8. J Immunol Meth.

[27] Fraser DC, Craigmile S, Campbell JD (1996). Functional expression of a cattle MHC class II DR-like antigen on mouse L cells. Immunogenetics.

[28] Goddeeris BM, Morrison WI, Teale AJ, Bensaid A, Baldwin CL (1986). Bovine cytotoxic T-cell clones specific for cells infected with the protozoan parasite *Theileria parva*: parasite strain specificity and class I major histocompatibility complex restriction. Proc Natl Acad Sci USA.

[29] Morrison WI, Goddeeris BM, Teale AJ (1987). Cytotoxic T-cells elicited in cattle challenged with *Theileria parva* (Muguga): evidence for restriction by class I MHC determinants and parasite strain specificity. Parasite Immunol.

[30] Gillespie GM, Wills MR, Appay V (2000). Functional heterogeneity and high frequencies of cytomegalovirus-specific CD8 (+) T lymphocytes in healthy seropositive donors. J Virol.

[31] Sandberg JK, Fast NM, Nixon DF (2001). Functional heterogeneity of cytokines and cytolytic effector molecules in human CD8+ T lymphocytes. J Immunol.

[32] Aandahl EM, Sandberg JK, Beckerman KP (2003). CD7 is a differentiation marker that identifies multiple CD8 T cell effector subsets. J Immunol.

[33] Ellis SA, Holmes EC, Staines KA (1999). Variation in the number of expressed MHC genes in different cattle class I haplotypes. Immunogenetics.

[34] Townsend AR, McMichael AJ (1985). Specificity of cytotoxic T lymphocytes stimulated with influenza virus. Studies in mice and humans. Prog Allergy.

[35] Vitiello A, Sherman LA (1983). Recognition of influenza-infected cells by cytolytic T lymphocyte clones: determinant selection by class I restriction elements. J Immunol.

